# Molten salt electro‐preparation of graphitic carbons

**DOI:** 10.1002/EXP.20210186

**Published:** 2023-01-09

**Authors:** Fei Zhu, Jianbang Ge, Yang Gao, Shijie Li, Yunfei Chen, Jiguo Tu, Mingyong Wang, Shuqiang Jiao

**Affiliations:** ^1^ School of Metallurgical and Ecological Engineering University of Science and Technology Beijing Beijing China; ^2^ State Key Laboratory of Advanced Metallurgy University of Science and Technology Beijing Beijing China; ^3^ Beijing Key Laboratory of Green Recycling and Extraction of Metals University of Science and Technology Beijing Beijing China; ^4^ Institute of Advanced Structure Technology Beijing Institute of Technology Beijing China

**Keywords:** amorphous carbon, CO_2_ conversion, molten salt electrolysis, molten salt graphitization, synthetic graphite

## Abstract

Graphite has been used in a wide range of applications since the discovery due to its great chemical stability, excellent electrical conductivity, availability, and ease of processing. However, the synthesis of graphite materials still remains energy‐intensive as they are usually produced through a high‐temperature treatment (>3000°C). Herein, we introduce a molten salt electrochemical approach utilizing carbon dioxide (CO_2_) or amorphous carbons as raw precursors for graphite synthesis. With the assistance of molten salts, the processes can be conducted at moderate temperatures (700–850°C). The mechanisms of the electrochemical conversion of CO_2_ and amorphous carbons into graphitic materials are presented. Furthermore, the factors that affect the graphitization degree of the prepared graphitic products, such as molten salt composition, working temperature, cell voltage, additives, and electrodes, are discussed. The energy storage applications of these graphitic carbons in batteries and supercapacitors are also summarized. Moreover, the energy consumption and cost estimation of the processes are reviewed, which provides perspectives on the large‐scale synthesis of graphitic carbons using this molten salt electrochemical strategy.

## Graphical abstract

This review shows that CO_2_ gas or biomass‐derived carbons could be utilized as raw precursors for the preparation of graphitic carbons via a molten salt electrochemical route. In this review, we discuss the factors that affect the graphitization, the costs of the processes, and the challenges that need to be tackled. The molten salt approach has shown great promise in large‐scale synthesis of high‐quality graphite materials in future.

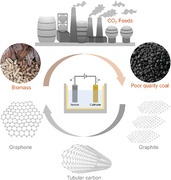

## 1 INTRODUCTION

Graphite has a layer structure in which the atoms are arranged in a hexagonal or HCP crystal pattern and the layers are stacked in the AB sequence. The layered pattern of graphite results in its extreme anisotropy and the carbon layers can easily slide, which makes graphite a soft material and an excellent lubricant. Besides, graphite possesses great chemical stability, excellent electrical conductivity, and outstanding mechanical properties, which enables graphite to be widely used in modern society for diverse applications (e.g., batteries, electric brushes, crucibles, and refractories).^[^ ^1^, ^2^, ^3^, ^4^ ^]^

The widely used graphite with rising demand impels the continuous development of synthetic graphite.^[^ ^5^, ^6^ ^]^ Synthetic graphite was first made by Edward G. Acheson at the end of the 19th century. The process involved two steps: (1) the chemical reaction between coke and silicon oxide to form silicon carbide (SiC,∼2000°C); (2) the vaporization of silicon from SiC at an ultra‐high temperature of 4200°C.^[^ ^7^, ^8^ ^]^ Later, it was demonstrated that the graphitization can be established by direct high‐temperature treatment.^[^ ^6^ ^]^ To date, synthetic graphite is mainly prepared by the high‐temperature treatment (∼3000°C) of calcined petroleum coke,^[^ ^9^ ^]^ which is energy‐intensive.^[^ ^6^, ^8^ ^]^

Multiple approaches employed at moderate temperatures have been adopted to reduce the energy consumption. It has been reported that adding transition metals (e.g., nickel (Ni) and iron (Fe)) into cokes lowers the working temperature (1000–1400°C).^[^ ^10^, ^11^, ^12^, ^13^ ^]^ The added transition metal will react with carbon (C) to form metal carbides, which will further decompose into graphitic carbon and transition metal upon a higher temperature; that is, the transition metals are served as catalysts for the graphitization process. Moreover, microwave heating can also be introduced to assist this catalytic graphitization process.^[^ ^14^ ^]^ The catalytic graphitization process has been extensively studied and the main challenge that limits its future development is the removal of metal carbide impurities encapsulated in the graphite products. Chemical vapor deposition (CVD) is another approach for producing graphite by the thermal decomposition of hydrocarbon gases and the deposition of carbon.^[^ ^15^ ^]^ However, the low yield and high cost make it impossible for the large‐scale synthesis of graphite. Recently, molten salt electro‐preparation of graphitic carbon materials has become an effective approach owing to the low working temperature, simplicity, and fast reaction kinetics.^[^ ^16^, ^17^ ^]^ In this approach, graphitic carbon materials can be obtained by either electrochemical reduction of CO_2_ or electrochemical graphitization of amorphous carbons.

Molten salt electrochemical conversion of CO_2_ has been demonstrated to be a promising approach for reducing CO_2_ emissions in which CO_2_ can be efficiently split into carbon (at cathode) and oxygen gas (at an inert anode) in molten salt systems.^[^ ^18^, ^19^, ^20^, ^21^, ^22^, ^23^ ^]^

This CO_2_ conversion process involves two steps: (1) the absorption of CO_2_ by the dissolved O^2−^ ions; (2) the reduction of the formed CO_3_^2−^ ions into carbon and oxygen gas.^[^ ^24^, ^25^, ^26^, ^27^, ^28^, ^29^, ^30^, ^31^, ^32^, ^33^, ^34^ ^]^ The most frequently used molten salt systems are molten carbonates and molten chlorides, where the solubility of O^2−^ ions are relatively high. Note that most fluorides (e.g., sodium fluoride, lithium fluoride, and potassium fluoride) have limited solubility in water and are difficult to remove. Therefore, only a few literatures studied the electroreduction of CO_2_ in molten fluorides and will not be included in the present review.^[^ ^35^, ^36^ ^]^ More information on the thermodynamics and kinetics of CO_2_ absorption in molten salts can be found in previous studies.^[^ ^18^, ^19^, ^20^ ^]^ In general, the electrochemical reduction of CO_3_^2‐^ is the rate‐determining process and determines the properties of the deposited carbon.^[^ ^18^ ^]^ Besides, amorphous carbons, including coke, carbon black, and carbon fibers, can also be directly converted into graphite through cathodic polarization.^[^ ^37^, ^38^, ^39^ ^]^ Several reviews on the electro‐splitting of CO_2_ into carbon materials in molten salts have been reported.^[^ ^40^, ^41^, ^42^, ^43^, ^44^ ^]^ However, the obtained products are amorphous carbons in many cases and a thorough summary of the preparation of graphitic carbons is essential for a deeper understanding of the conversion mechanism.

In this review, we focus on the electro‐preparation of graphitic carbons in molten salts, using CO_2_ and amorphous carbons as carbon resources. The underlying reaction mechanisms that govern the electrochemical processes are presented. Considering the physiochemical properties and microstructures of the carbon products derived from CO_2_ are highly dependent on the salt system, the preparation of graphitic carbons in molten carbonates and chlorides is discussed, respectively. Moreover, the effects of electrolytic parameters, such as working temperature, cell voltage, additives, and electrodes, on the graphitization degree of the obtained carbon materials are analyzed. The applications of these graphitic carbons in energy storage are highlighted. Last, the energy consumption and cost estimation of this molten salt approach are provided to evaluate the potential for further commercialization.

## 2 ELECTROCHEMICAL CONVERSION OF CO_2_ INTO GRAPHITIC CARBONS

The conversion of CO_2_ gas in molten salts can proceed at an appreciable reaction rate compared to those in aqueous solution and room temperature ionic liquids.^[^ ^45^, ^46^, ^47^, ^48^ ^]^ Carbon products can be easily deposited in this aprotic salt system. However, the obtained carbon products contain large amounts of amorphous carbon.^[^ ^27^, ^28^, ^29^, ^30^, ^49^, ^50^, ^51^, ^52^, ^53^, ^54^, ^55^, ^56^ ^]^ An increased temperature can only slightly enhance the graphitization degree of the obtained carbon.^[^ ^51^, ^53^ ^]^ In order to convert the amorphous carbons into graphitic carbons, small amounts of additives or catalysts are usually needed, depending on the salt system. The electrolytic parameters such as current density or cell voltage, electrolysis time, and electrode material should be also well controlled to obtain graphite with high quality and well crystallinity. The electrolytic conditions for preparing graphitic carbons have been summarized in Tables 1 and 2 for comparison.

**TABLE 1:** The collection of the energy consumption of CO_2_ electrochemical conversion in molten carbonates

**Electrolyte**	**Cathode**	**Anode**	**T (°C)**	**Cell voltage (V) or current density (A/cm^2^)**	**Additive or catalyst in salt**	**Additional operation**	**Energy consumption (kWh/kg C)**	**Dominated products**	**Ref**.
Li_2_CO_3_	Ni/steel/Pt	Pt/Ir	750–900	0.1–1 A/cm^2^	N/A	N/A	8.04–15.63	Amorphous carbon	^[^ ^57^, ^58^, ^59^ ^]^
Li_2_CO_3_	Zn coated steel	Ir	770	0.1–1 A/cm^2^	Low NiO or Fe_2_O_3_	N/A	8.04–15.63	Amorphous carbon	^[^ ^58^ ^]^
Li_2_CO_3_	Zn coated steel	Ir	770	0.1–1 A/cm^2^	low NiO or Fe_2_O_3_	Initial nucleation	8.04–15.63	Straight CNFs	^[^ ^58^ ^]^
Li_2_CO_3_	Zn coated steel	Ir	770	0.1–1 A/cm^2^	Li_2_O, low NiO or Fe_2_O_3_	Initial nucleation	8.04–15.63	Tangled CNFs	^[^ ^58^ ^]^
Li_2_CO_3_	Zn coated steel	Ni or NiCr	770	0.1–1 A/cm^2^	N/A	Initial nucleation	∼10.72^a^	Straight CNTs	^[^ ^60^, ^61^ ^]^
Li_2_CO_3_	Zn coated steel	Ni	770	0.1–1 A/cm^2^	^13^CO_2_ gas	Initial nucleation	8.04–15.63	Straight CNFs	^[^ ^62^ ^]^
Li_2_CO_3_	Zn coated steel	Ni	770	0.1–1 A/cm^2^	Li_2_O	Initial nucleation	8.04–15.63	Tangled CNTs	^[^ ^63^ ^]^
Li_2_CO_3_	Zn coated steel	Ni	770	0.1–1 A/cm^2^	LiBO_2_, LiPO_3_, or LiNO_3_	Initial nucleation	8.04–37.5^a^	CNTs	^[^ ^64^ ^]^
Li_2_CO_3_	Monel	Ni	770	0.1–1 A/cm^2^	Ni particles	N/A	∼10.72^a^	CNTs	^[^ ^60^ ^]^
Li_2_CO_3_	Monel	NiCr	770	0.1–1 A/cm^2^	N/A	N/A	∼10.72^a^	Long CNT wool	^[^ ^60^ ^]^
Li_2_CO_3_	Ni‐coated Cu	Ir	770	0.1–1 A/cm^2^	N/A	N/A	9.8–12.5^a^	CNTs	^[^ ^65^ ^]^
Li_2_CO_3_	Steel, brass, Fe coated steel, or ZnO coated Fe	Passivated Ni	750	0.025–0.1 A/cm^2^	N/A	N/A	N/A	CNTs	^[^ ^66^, ^67^, ^68^, ^69^ ^]^
Li_2_CO_3_	Zn coated Cu	Ir	770	0.1–1 A/cm^2^	Li_2_O	N/A	9.8–12.5^a^	CNOs	^[^ ^65^ ^]^
Li_2_CO_3_─M*_x_* CO_3_ (M = K, Na, *x* = 2)	Ni, Fe, or graphite	Graphite	500–700	0.15–1.2 A/cm^2^	N/A	N/A	41.22–54.97^a^	Amorphous carbon	^[^ ^49^, ^51^, ^70^ ^]^
Li_2_CO_3_─Na_2_CO_3_─K_2_ CO_3_─MCO_3_ (M = Ca, Sr, Ba), Na_2_CO_3_─K_2_CO_3_─CaCO_3_	Ni or Fe	NiCuFe or SnO_2_	450–650	0.05–0.2 A/cm^2^ or 3.0–6.0 V	N/A	N/A	20.6–82.51	Amorphous carbon	^[^ ^71^, ^72^, ^73^ ^]^
Li_2_CO_3_─Na_2_CO_3_─K_2_CO_3_─MCO_3_ (M = Ca, Sr, Ba)	Zn coated Steel	Ni	600	0.25 A/cm^2^	N/A	Initial nucleation	N/A	Low yield CNTs	^[^ ^74^ ^]^
Li_2_CO_3_─M*_x_* CO_3_ (M = Na,K, *x* = 2; M = Sr, Ba, Ca, *x* = 1)	Zn coated steel	Ni	730–770	0.05–1 A/cm^2^	N/A	Initial nucleation	9.6–35.7^a^	CNTs	^[^ ^74^, ^75^, ^76^, ^77^ ^]^
Li_2_CO_3_─CaCO_3_─BaCO_3_	Zn coated steel	Ni	750	0.2 A/cm^2^	N/A	Initial nucleation	N/A	80% CNTs	^[^ ^76^ ^]^
Na_2_CO_3_─K_2_CO_3_	Ni	SnO_2_	800	3.0–4.0 V	NaVO_3_	N/A	N/A	Graphite	^[^ ^78^ ^]^
Li_2_CO_3_─Na_2_CO_3_─K_2_CO_3_	Ni	SnO_2_	775	0.0625 A/cm^2^	Li_2_SO_4_	N/A	N/A	Graphite	^[^ ^25^ ^]^

^a^ The value is estimated based on the data in Refs.

**TABLE 2:** The collection of the energy consumption of CO_2_ electrochemical conversion in molten chlorides

**Electrolyte**	**Cathode**	**Anode**	***T* (°C)**	**Cell voltage (V) or current (A)**	**Additive or catalyst in salt**	**Energy consumption (kWh/kg C)**	**Dominated products**	**Ref**.
CaCl_2_─CaO	Steel	RuO_2_·TiO_2_	850	2.6 V	N/A	N/A	Graphite sheet	^[^ ^27^ ^]^
CaCl_2_─NaCl─CaO	Steel or glass carbon	RuO_2_·TiO_2_	750	2.2–2.8 V	N/A	N/A	Graphene or CNTs	^[^ ^28^, ^29^ ^]^
CaCl_2_─LiCl─CaO	Cu or Ni	SnO_2_	600	3.0 V	N/A	N/A	Amorphous carbon	^[^ ^33^ ^]^
CaCl_2_─LiCl─Na_2_CO_3_, CaCl_2_─Na_2_CO_3_	Steel	Graphite	850	3.0 V	N/A	N/A	Amorphous carbon	^[^ ^34^ ^]^
LiCl─KCl─CaCl_2_─CaCO_3_	Steel	SnO_2_	500–800	3.0–4.0 V	N/A	28.2–37.6	Amorphous carbon	^[^ ^79^, ^80^ ^]^
LiCl─NaCl─Na_2_CO_3_	W	Pt	650–800	2.2–2.8 V	N/A	20.1–49.1	Amorphous carbon	^[^ ^53^ ^]^
LiCl─KCl─CaCO_3_	Ni	Graphite	450–650	2.8–4.5 V	N/A	29.4–87.9^a^	Amorphous carbon	^[^ ^52^ ^]^
CaCl_2_─CaCO_3_	Ni	Graphite	850	∼3.0 V	B_2_O_3_	N/A	CNTs	^[^ ^32^ ^]^
CaCl_2_─NaCl─CaO	Graphite	RuO_2_·TiO_2_	750	0.75 A	Li_2_SO_4_	44.06–129.53	Graphite sheet or CNTs	^[^ ^30^ ^]^
CaCl_2_─NaCl─Na_2_SO_4_─CaO	Graphite	RuO_2_·TiO_2_	750	0.75 A	NiO, Fe_2_O_3_ or Cr_2_O_3_	47.5–104.3	CNFs	^[^ ^31^ ^]^

^a^ The value is estimated based on the data in Refs.

### 2.1 Electrochemical reduction of CO_2_ in molten salts

**FIGURE 1:**
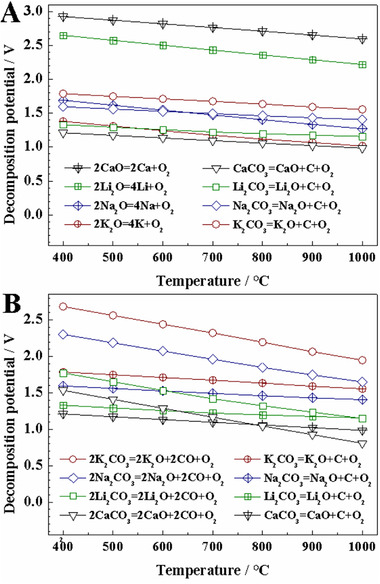
(A) Thermodynamic calculation of the decomposition voltages for alkali metals deposition and carbon deposition. (B) Thermodynamic calculation of the decomposition voltages for CO evolution and carbon deposition. The calculations are conducted assuming all the components are in standard state. Data from HSC software.

The electrochemical reduction of CO_2_ is mainly carried out in either molten carbonates or chlorides because of their high solubility of O^2−^ ions. In molten carbonates, most studies are carried out in molten alkali metal carbonates lithium carbonate (Li_2_CO_3_), sodium carbonate (Na_2_CO_3_), and potassium carbonate (K_2_CO_3_)) due to their superior stabilities under high working temperatures.^[^ ^24^, ^25^, ^26^, ^71^, ^72^, ^73^, ^74^, ^79^, ^80^, ^81^, ^82^, ^83^, ^84^, ^85^ ^]^ In terms of thermodynamics (data from HSC chemistry), Li_2_CO_3_ is an essential component in molten carbonates for carbon deposition in Li_2_CO_3_─Na_2_CO_3_─K_2_CO_3_ system, as shown in Figure 1A. The decomposition potential of potassium oxide (to potassium metal) is much lower than that of carbon deposition reaction, and the decomposition potential of sodium oxide is close to that of carbon deposition. As a result, in either molten Na_2_CO_3_ or K_2_CO_3_, alkali metal deposition occurs more readily than carbon deposition (reaction 1), which has been experimentally confirmed.^[^ ^54^ ^]^ In contrast, lithium oxide (Li_2_O) and calcium oxide (CaO) with high decomposition potentials show excellent electrochemical stability in molten carbonate and can be served as the adsorbents for CO_2_. The calculation also implies that carbon deposition is preferred in molten calcium carbonate (CaCO_3_). However, CaCO_3_ is unstable and decomposes at 825°C. Magnesium carbonate (MgCO_3_) is even worse and decomposes at 350°C. Other carbonates such as barium carbonate (BaCO_3_) are stable but expensive.^[^ ^79^ ^]^ Therefore, Li_2_CO_3_‐based carbonates are commonly used as supporting electrolytes for carbon deposition. CaCO_3_ and BaCO_3_ are selected as additives in most cases. In molten chlorides, considering the relatively high solubility of O^2−^ in molten calcium chloride (CaCl_2_) and lithium chloride (LiCl), investigations on carbon deposition are usually conducted in CaCl_2_‐based and LiCl‐based chloride salts.^[^ ^19^ ^]^

(1) $$C{O_{3}}^{2-}+4e\to C+3{O^{2}}^{-}$$

(2) $$C{O_{3}}^{2-}+2e\to \mathrm{CO}+2{O^{2}}^{-}$$

The competing reaction for carbon deposition in molten salts is the evolution reaction of carbon monoxide (CO, reaction 2). As shown in Figure 1B, CO evolution reaction is thermodynamically easier than carbon deposition when working temperature is higher than 970°C in molten Li_2_CO_3_ (all the thermodynamic calculations are established assuming the activity of all components is unity). Licht et al. have demonstrated that the content of CO product increased with the working temperature in molten Li_2_CO_3_.^[^ ^57^ ^]^ The molar ratios of CO/C at 750°C, 850°C and 900°C were 0.05, 0.5 and 2, respectively. CO gas was the sole product at 950°C. Kaplan et al. reported the successful conversion of CO_2_ to CO in molten Li_2_CO_3_ at 900°C, with a faradaic efficiency close to 100%.^[^ ^86^ ^]^ The evolution of CO occurred at a temperature much lower than the calculated 970°C due to the low partial pressure of CO gas during experiments. Chery et al. made a thermodynamic study on the electrochemical reduction of CO_2_ into CO or C in carbonates (Li_2_CO_3_─K_2_CO_3_, Li_2_CO_3_─Na_2_CO_3_, Na_2_CO_3_─K_2_CO_3_, and Li_2_CO_3_─Na_2_CO_3_─K_2_CO_3_).^[^ ^87^, ^88^, ^89^ ^]^ The parameters affecting the final products, such as temperature, gas partial pressures, and molten carbonates, were comprehensively discussed. It has been concluded that high temperature, high CO_2_ partial pressure, and low CO partial pressure were favorable for the CO production, while low temperature, high CO_2_ partial pressure, and high CO partial pressure facilitated the carbon deposition. Intriguingly, no CO was detected in LiCl─Li_2_CO_3_ system even at 900°C, implying that carbon deposition was dominate in molten chlorides.^[^ ^53^, ^55^ ^]^ Besides, the thermodynamic calculation shows that CO evolution reaction is more preferable at temperatures higher than 800°C for CaCO_3_ decomposition. Matsuura et al. demonstrated the high yield electro‐splitting of CO_2_ into CO gas in CaCl_2_─CaO salt at 900°C.^[^ ^88^ ^]^ Hu et al. observed the appreciable evolution of CO gas in CaCl_2_─NaCl─CaO bubbled with CO_2_ at a cell voltage of 2.8 V and 750°C.^[^ ^28^, ^29^ ^]^ Deng et al. concluded that CO evolution could occur in molten LiCl─KCl─CaCO_3_ at temperatures as low as 450°C, even though no direct proof of CO evolution was provided.^[^ ^52^ ^]^

**FIGURE 2:**
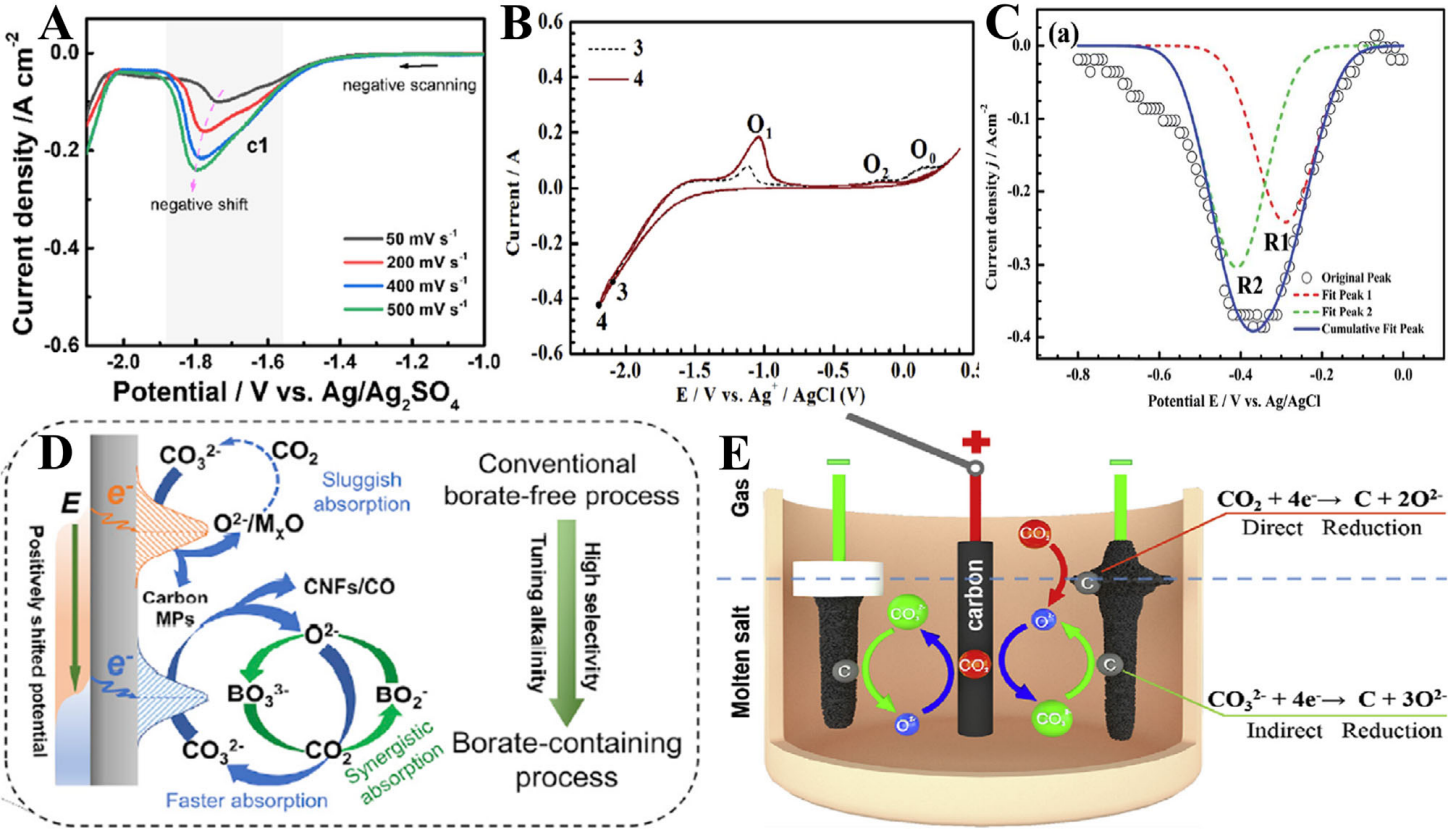
(A) Linear sweep voltammetry recorded on a Ni electrode in molten Li_2_CO_3_─Na_2_CO_3_─K_2_CO_3_ under CO_2_ atmosphere at 450°C. Reproduced with permission.^[^ ^26^ ^]^ Copyright 2021, Elsevier. (B) Cyclic voltammograms recorded on a glass carbon electrode in LiCl─NaCl─Na_2_CO_3_ under Ar atmosphere at 750°C. Reproduced with permission.^[^ ^53^ ^]^ Copyright 2016, Elsevier. (C) Square wave voltammogram recorded on a W electrode in molten CaCl_2_─CaCO_3_ at 850°C. Reproduced with permission.^[^ ^27^ ^]^ Copyright 2015, Royal Society of Chemistry. (D) The buffering effect of LiBO_2_ on tunable production for CNFs or CO in LiCl─Li_2_CO_3_ system at low temperature (<650°C). Reproduced with permission.^[^ ^91^ ^]^ Copyright 2020, Cell Press. (E) The growth of carbon on an electrode through direct and indirect reduction of CO_2_ in molten CaCl_2_─NaCl─CaCO_3_. Reproduced with permission.^[^ ^92^ ^]^ Copyright 2019, Elsevier.

For the reduction of CO_3_^2−^ into carbon, it is generally accepted that there are two pathways, single step reduction and two step reduction.^[^ ^26^, ^28^, ^29^, ^53^, ^79^, ^80^, ^90^, ^91^ ^]^ The single step reduction involves a direct four‐electron transfer (reaction 1), which has been validated by low temperature electrolysis. No CO gas was detected in the exhaust gases during the electrolysis in Li_2_CO_3_─Na_2_CO_3_─K_2_CO_3_ eutectic at 450°C, and large amounts of carbon deposits were observed at the cathode, confirming the single step reduction mechanism.^[^ ^54^ ^]^ The peak c1 in Figure 2A in cyclic voltammetry (CV) tests refers to carbon deposition, and the peak potential is negatively shifted as the scan rate increases, showing a diffusion‐controlled and quasi‐reversible process.^[^ ^26^ ^]^ The oxidation peaks in Figure 2B, O_1_ and O_2_, represent the oxidation of the carbon products, as given by reactions (3) and (4).^[^ ^53^ ^]^ The two‐step reduction process involves the reduction of CO_3_^2−^ to hypothetical CO_2_^2−^ ions (reaction 5) and subsequent reduction of CO_2_^2−^ ions to C (reaction 6). The hypothetical CO_2_^2−^ ion is unstable and will decompose into CO gas and O^2−^ ion (reaction 7).^[^ ^28^, ^29^, ^86^ ^]^ The two‐step reduction mechanism was simply verified by square wave voltammetry (SWV, Figure 2C).^[^ ^27^, ^86^ ^]^ The peak R1 and peak R2 are related to reactions (5) and (7), respectively.^[^ ^27^ ^]^ However, this conclusion might be not rigorous as the intermediate species, CO_2_^2−^, was not experimentally detected by any in situ techniques (e.g., in situ NMR and Raman spectroscopy). Moreover, the quantitative analysis of the reaction kinetics (both carbon deposition and CO evolution) is remaining a challenge owing to the harsh environment.

(3) $$C+2{O^{2}}^{-}\to CO_{2}+4e$$

(4) $$C+2C{O_{3}}^{2-}\to 3CO_{2}+4e$$

(5) $$C{O_{3}}^{2-}+2e\to C{O_{2}}^{2-}+{O^{2}}^{-}$$

(6) $$C{O_{2}}^{2-}+2e\to C+2{O^{2}}^{-}$$

(7) $$C{O_{2}}^{2-}↔\mathrm{CO}+{O^{2}}^{-}$$

The CO_2_ reduction in molten salt can be tuned by regulating the activity of O^2−^. Hu et al. have reported the “buffer effect” provided by borate (BO_2_^−^) ions in molten LiCl─Li_2_CO_3_ (Figure 2D).^[^ ^90^, ^91^ ^]^ The added BO_2_^−^ ions can absorb O^2−^ ions to form BO_3_^3−^, which significantly decrease the activity of O^2−^ (reaction 8). The formed BO_3_^3‐^ ions can then react with CO_2_ to regenerate BO_2_^−^ and release CO_3_^2−^ (reaction 9). The decreased activity of O^2−^ positively shifts the potentials of carbon deposition and CO evolution, which allows the reactions to occur at low temperatures. With the assistance of 5 mol% lithium borate (LiBO_2_), the carbon deposition with a current efficiency of ∼93% was confirmed at 550°C, while the CO evolution dominated at 650°C and a current density of 100 mA/cm^2^.

(8) $$B{O_{2}}^{-}+O^{2-}↔B{O_{3}}^{3-}$$

(9) $$B{O_{3}}^{3-}+CO_{2}↔B{O_{2}}^{-}+C{O_{3}}^{2-}$$

Apart from the reduction of CO_3_^2−^ ions, it has been demonstrated that CO_2_ gas can also be directly reduced into carbon at the “CO_2_/molten salt/solid electrode” interface (reaction 10), as shown in Figure 2E.^[^ ^92^ ^]^ The preferential growth of carbon products along the interface can be promoted or hindered by controlling the gas atmosphere and temperature. Generally, electrolysis under CO_2_ atmosphere and high temperatures (>700°C) can greatly enhance the carbon growth.

(10) $$CO_{2}+4e\to C+2{O^{2}}^{-}$$

The application of inert anodes that enable the O_2_ evolution is of great importance in the electrochemical reduction of CO_2_ into carbon materials (reactions 11 and 12).^[^ ^27^, ^28^, ^29^, ^93^, ^94^, ^95^, ^96^, ^97^, ^98^ ^]^ Thus, the overall reaction can be described as the splitting of CO_2_ into C and O_2_ gas (reaction 13). The stabilities of inert anodes strongly depend on the anode material, molten salt composition, and working temperature. In molten carbonates, it has been demonstrated that Ni‐based anodes showed limited corrosion and could maintain stable O_2_ evolution for over 24 h.^[^ ^94^ ^]^ In molten chlorides, several types of anode materials, including ceramics, cermet, and alloys, have been considered, however, none of them could demonstrate the feasibility for long‐time tests.^[^ ^98^, ^99^ ^]^

(11) $$2{O^{2}}^{-}-4e\to O_{2}$$

(12) $$3C{O_{3}}^{2-}-4e\to O_{2}+3CO_{2}+{O^{2}}^{-}$$

(13) $$CO_{2}↔C+O_{2}$$

### 2.2 Preparation of graphitic carbons in molten carbonates

The presence of Li_2_CO_3_ in molten carbonates is essential for carbon deposition due to its excellent stability and wide electrochemical window, allowing carbon deposition to take place at a high current density. Therefore, a series of studies were first conducted in molten Li_2_CO_3_ for preparing graphitic carbons. Licht et al. demonstrated the high yield synthesis of carbon nanofibers (CNFs) and carbon nanotubes (CNTs) in Li_2_CO_3_ by depositing trace amounts of transition metals, particularly Ni, as catalysts onto the cathode surface. The isolated transition metal atoms deposited at the cathode could serve as nucleation sites for the growth of CNFs or CNTs. Furthermore, the length and diameter of the CNFs and CNTs can be controlled by adjusting the electrolytic conditions, such as the concentration of catalyst in salt, the treatment of electrodes, and the control of current density.^[^ ^57^, ^58^, ^59^, ^60^, ^62^, ^63^, ^64^, ^65^, ^75^, ^100^ ^]^

**FIGURE 3:**
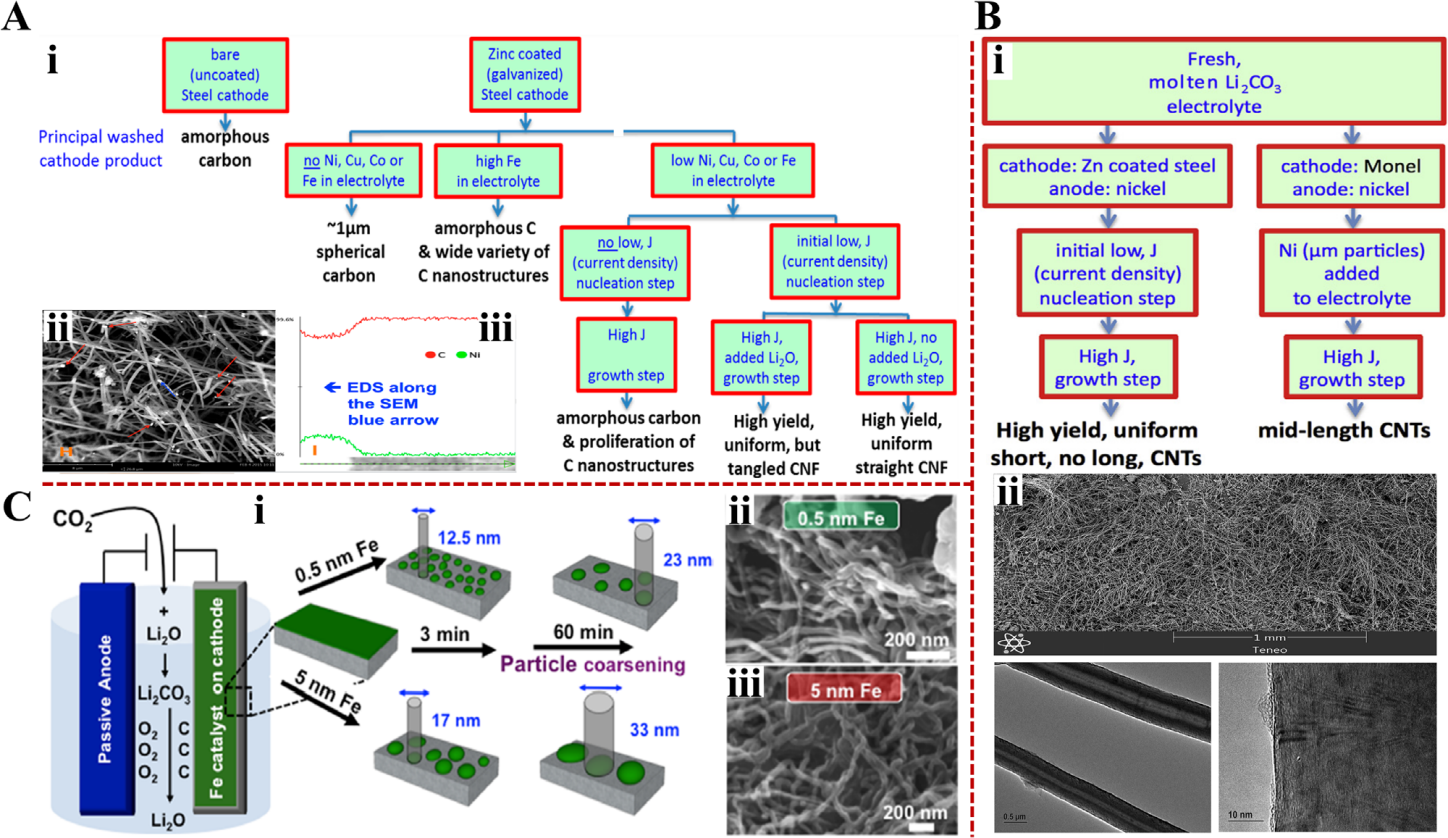
(A) Electrochemical conversion of CO_2_ into CNFs in molten Li_2_CO_3_: (i) electrolytic pathways for preparing high quality CNFs; SEM image (ii) and EDS analysis (iii) of the obtained CNF product. Reproduced under the terms of the ACS AuthorChoice License.^[^ ^58^ ^]^ Copyright 2015, American Chemical Society. (B) Electrochemical conversion of CO_2_ into CNTs in molten Li_2_CO_3_: (i) electrolytic pathways for preparing CNTs; (ii) SEM image of the obtained CNT wool. Reproduced with permission.^[^ ^60^ ^]^ Copyright 2017, Elsevier. (C) The effect of Fe layer thickness on the CNT growth: (i) schematic illustration of electrolysis setup and the control of the CNT diameter by varying Fe layer thickness in molten Li_2_CO_3_; SEM images of the CNT growth from 0.5 nm Fe layer (ii) and 5 nm Fe layer (iii). Reproduced with permission.^[^ ^67^ ^]^ Copyright 2018, American Chemical Society.

The direct addition of small amounts of transition metal oxides into Li_2_CO_3_ has been proved a feasible way to achieve CNFs through electrolysis. Figure 3A(i) shows the pathways that transform CO_2_ into high yield CNFs products. As expected, amorphous carbon is the sole product under the electrolysis between an iridium (Ir) anode and a steel cathode in pure Li_2_CO_3_. The preparation of CNFs requires a precise control of electrolytic condition. Only with small amounts of dissolved transition metal oxides (e.g., nickel oxide (NiO)) and an initial nucleation step, can CNFs be deposited at a Zinc‐coated (Zn‐coated) steel cathode. The initial nucleation step, which usually refers to an electrolysis conducted at 5 mA/cm^2^ for 1 h prior to carbon deposition, is carried out for the deposition of the transition metals. Note that the current density applied in this step is usually limited to a range of 5 to 10 mA/cm^2^, and a high current density (>20 mA/cm^2^) incurs the formation of amorphous carbon. The deposited transition metal, such as Ni, can then act as nucleation sites for the growth of CNFs. Further scanning electron microscopy (SEM) and energy dispersive spectroscopy (EDS) analysis of the obtained CNFs (Figure 3A(ii and iii)) reveal that Ni metal is located at the tip (or bottom) of the CNFs, proving that Ni can be served as nucleation site to induce the CNF growth. However, an excess addition of transitions metal oxides into salt results in the formation of amorphous carbon and a wide variety of carbon nanostructures, implying the aggregation of large‐diameter CNFs.^[^ ^61^, ^66^ ^]^ Besides, the Zinc (Zn) coated steel plays an essential role for CNF growth because Zn coating can greatly lower the carbon forming energy.^[^ ^65^ ^]^ In the absence of this coating, the products are comprised of amorphous carbon and non‐uniform CNFs even with the NiO or iron oxide (Fe_2_O_3_) additive.

The employment of Ni or nickel‐chromium alloy anode, instead of a typical inert Ir anode, is another strategy for introducing Ni into molten salt, as illustrated in Figure 3B(i). Upon electrolysis, the Ni anode can maintain stable after the initial corrosion. The trace amounts of the dissolved Ni^2+^ will in turn be deposited at the Zn‐coated steel cathode to form the Ni nucleation sites and then to initiate the growth of CNTs. The attempt to remove Zn coating leads to the low yield CNTs, and an additional Ni particle in salt is needed to promote the CNT growth. Intriguingly, replacing the Ni anode with a NiChrome anode significantly facilitates the CNT synthesis, where a high‐quality CNT wool product with a length ranging from 0.4 to 1.2 mm is obtained owing to the synergistic nucleation effect from the deposited Ni and Cr atoms (Figure 3B(ii)).^[^ ^60^ ^]^

The transition metal can also be directly deposited at the cathode with controlled thickness for regulating the CNT growth (Figure 3C(i)). Douglas et al. have systematically studied the catalytic effect of Fe on the formation of CNTs.^[^ ^61^, ^66^, ^67^, ^68^, ^69^ ^]^ High yield multi‐walled CNTs could be obtained at a Fe or Zinc oxide‐coated Fe cathode by passivating Ni anode during electrolysis, demonstrating the catalytic effect of Fe for the CNT formation.^[^ ^66^ ^]^ Moreover, the influence of the Fe layer thickness on the CNT formation is studied.^[^ ^67^ ^]^ The median diameters of the obtained CNTs increases from 23 to 33 nm as the layer thickness increases from 0.5 to 5 nm (Figure 3C(ii),(iii)). On the basis of these findings, multiple cathode materials containing Fe, such as metal scrap materials (brass pipes and brass screws), galvanized steel, stainless steel, and 1010 steel, can be used for the production of multi‐walled CNTs.^[^ ^68^ ^]^ The yield of multi‐walled CNTs at the 1010 steel reaches 90%, and high‐quality CNT products are obtained at a Zinc oxide‐coated galvanized steel with a high yield of 99%.

Electrochemical conversion CO_2_ into CNTs or CNFs could be achieved in molten Li_2_CO_3_ with the assistance of transition metals and appropriate electrolytic conditions (e.g., the use of Zn‐coated steel cathode and low concentration of transition metal oxides in the salt). It should be emphasized that high‐yield and high‐quality CNTs (or CNFs) are commonly obtained with a proper electrolysis time (2 h at a current density of 0.2 A/cm^2^, total charge is ∼0.4 Ah/cm^2^). A wide variety of carbon products are obtained under short‐time electrolysis (5–15 min),^[^ ^65^ ^]^ and the prolonged electrolysis (e.g., 20 h at 0.2 A/cm^2^) yields larger diameter CNTs (or CNFs) and a severe aggregation of CNTs (or CNFs).^[^ ^75^ ^]^ Additionally, a small area ratio of anode/cathode results in the accumulated O^2−^ ions in the salt, which can be electrochemically migrated to the cathode surface and then hinder the growth of the CNTs.^[^ ^69^ ^]^ A proper area ratio of anode/cathode should be selected for high quality CNT formation, depending on the applied anode. Besides, the electrolysis free of transition metals promotes the formation of carbon nanoonions.^[^ ^65^ ^]^

**FIGURE 4:**
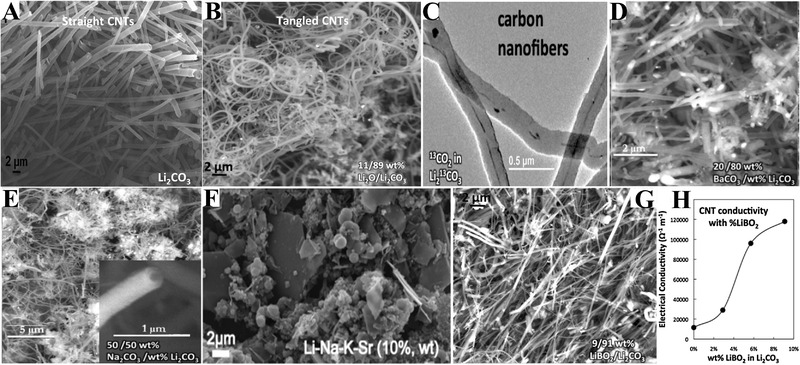
(A) SEM image of straight CNTs. Reproduced under the terms of the Creative Commons CC BY License.^[^ ^62^ ^]^ Copyright 2016, Nature Publishing Group. SEM images of tangled CNTs (B), CNFs derived from ^13^CO_2_ gas (C), CNTs produced in Li_2_CO_3_‐20 wt% BaCO_3_ (D), CNTs produced in Li_2_CO_3_‐50 wt% Na_2_CO_3_ (E), carbons produced in Li_2_CO_3_─Na_2_CO_3_─K_2_CO_3_‐10 wt% SrCO_3_ (F), and CNTs produced in Li_2_CO_3_‐9 wt% LiBO_2_ (G). (H) The electrical conductivity of CNTs obtained in Li_2_CO_3_ containing various concentration of LiBO_2_. Reproduced with permission.^[^ ^64^ ^]^ Copyright 2017, Elsevier.

In addition to the addition of transition metals (or oxides), the influence of other additives, such as Li_2_O, LiBO_2_, and non‐Li carbonates, on the carbon products has been studied as well. In general, the added Li_2_O results in the formation of tangled CNTs (or CNFs), while straight CNTs (or CNFs) are deposited at the cathode in the absence of Li_2_O (Figure 4A,B).^[^ ^62^ ^]^ Interestingly, the introduction of the heavier ^13^CO_2_ leads to the formation of CNFs because of its slow diffusion (Figure 4C).^[^ ^63^, ^64^ ^]^

Considering the relatively high price of Li_2_CO_3_, other economic carbonates such as Na_2_CO_3_, BaCO_3_, and CaCO_3_ are added into Li_2_CO_3_ and their corresponding effects on the carbon products are investigated.^[^ ^59^, ^70^, ^74^, ^76^, ^77^, ^101^ ^]^ High quality CNT products are deposited with a yield of 80% in the Li_2_CO_3_─BaCO_3_ system (Figure 4D), while the electrolysis in Li_2_CO_3_─K_2_CO_3_ yields 50%–80% CNTs.^[^ ^76^ ^]^ The effect of Na_2_CO_3_ on CNT growth is more complicated. The addition of 9 wt% Na_2_CO_3_ produces a carbon deposit comprising of 85% CNTs. The increase of Na_2_CO_3_ concentration to 30 wt% results in a low yield of CNTs (∼30%). However, the electrolysis in 50/50 wt% Li_2_CO_3_─Na_2_CO_3_ yields > 85% CNTs with a more tangled structure compared to that from pure Li_2_CO_3_ (Figure 4E).^[^ ^75^ ^]^ The carbon products from Li_2_CO_3_─CaCO_3_ system are mainly comprised of amorphous carbon and yield only 15% CNT. Moreover, high yield (>80%) of CNTs can be obtained in Li_2_CO_3_─CaCO_3_─BaCO_3_ carbonates, while carbon spheres are more favorable in molten Li_2_CO_3_─CaCO_3_─Na_2_CO_3_ and Li_2_CO_3_─CaCO_3_─K_2_CO_3_.^[^ ^76^, ^101^ ^]^ Note that the electrolysis in the Li_2_CO_3_─Na_2_CO_3_─K_2_CO_3_ or Li_2_CO_3_─Na_2_CO_3_─K_2_CO_3_─MCO_3_ (M = Ba, Ca, Sr) system provides low yield (15–30%) CNTs (Figure 4F).^[^ ^74^ ^]^ Although the introduction of these non‐Li carbonates greatly reduces the salt cost, the cell voltage during electrolysis in these salts increases by 0.3 V to 0.8 V compared to that in pure Li_2_CO_3_. The cell voltage for the electrolysis of Li_2_CO_3_ ranges from 1.1 to 1.4 V at 750°C and current densities in range of 0–1 A/cm^2^, while the cell voltage in Li_2_CO_3_─Na_2_CO_3_ system is in the range of 1.4–2.2 at 0–1 A/cm^2^.^[^ ^59^ ^]^ In the BaCO_3_‐Na_2_CO_3_ system, a much high cell voltage (1.8–3.0 V at 0–1 A/cm^2^) can be observed. The increased cell voltage should be related to the slow diffusion of O^2−^ ions that causes an overpotential at the anode.

The attempts to dope CNTs with non‐metal element (such as B, S, N, or P) have been carried out by the direct addition of LiBO_2_, lithium phosphate (LiPO_3_), lithium sulfate (Li_2_SO_4_), or lithium nitrate (LiNO_3_) into Li_2_CO_3_. The B‐doped CNTs, which possess an enhanced electrical conductivity (10 times than that of the CNTs from pure Li_2_CO_3_), are achieved (Figure 4G,H).^[^ ^64^ ^]^ High quality P‐doped CNTs are prepared with the additive of LiPO_3_, while a low yield of 40% N‐doped CNTs is obtained with the LiNO_3_ additive. However, no carbon deposits forms in the Li_2_SO_4_‐containing eutectic.

Molten Li_2_CO_3_‐Na_2_CO_3_‐K_2_CO_3_ eutectic is one of the most commonly used carbonates for carbon deposition, of which the carbon structures are amorphous (at a temperature of close to 700°C).^[^ ^51^, ^54^, ^84^, ^99^ ^]^ However, the strategy that deposits trace amounts of Ni at the cathode is proved to be ineffective to yield high quality CNTs (or graphite) in this system.^[^ ^74^ ^]^ To address this issue, various methods have been proposed and conducted to obtain high quality graphitic carbons in molten Li_2_CO_3_‐Na_2_CO_3_‐K_2_CO_3_.

**FIGURE 5:**
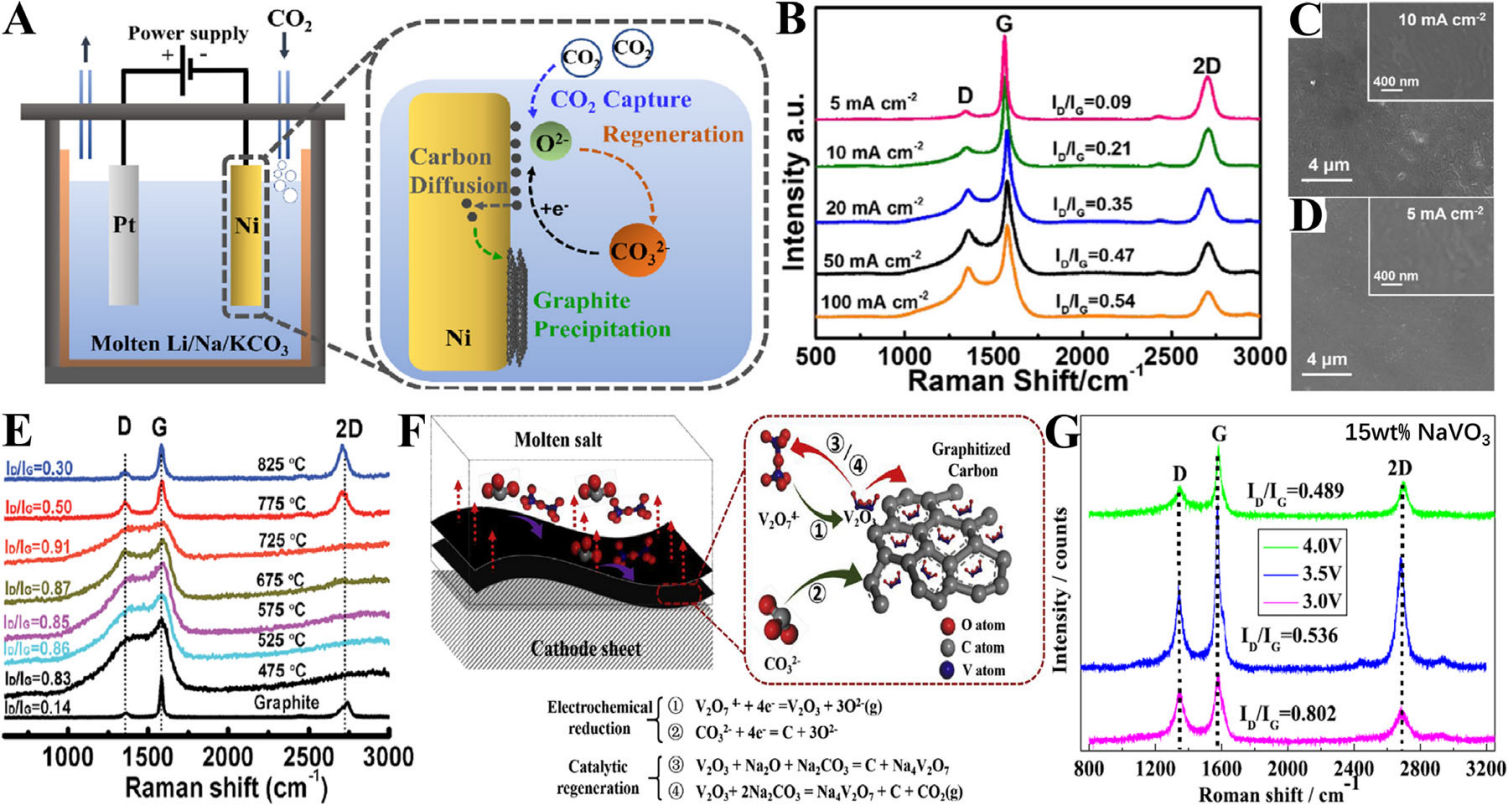
(A) Schematic illustrations of electrochemical synthesis of graphite in molten Li_2_CO_3_─Na_2_CO_3_─K_2_CO_3_; (B) Raman spectrums of the products deposited at various current densities; SEM images of the carbon deposited at 5 mA/cm^2^ (C) and 10 mA/cm^2^ (D), respectively. Reproduced with permission.^[^ ^26^ ^]^ Copyright 2021, Elsevier. (E) Raman spectrums of the carbon products obtained in molten Li_2_CO_3_─Na_2_CO_3_─K_2_CO_3_─Li_2_SO_4_ under different temperatures. Reproduced with permission.^[^ ^25^ ^]^ Copyright 2017, Royal Society of Chemistry. (F) Schematic illustration of the electrochemical conversion of CO_2_ into graphite in molten Na_2_CO_3_‐ K_2_CO_3_ under the in situ homogeneous catalysis; (G) Raman spectrums of the obtained carbon products under different electrolysis time (cell voltage: 3.5 V; NaVO_3_ concentration: 15 wt%). Reproduced with permission.^[^ ^78^ ^]^ Copyright 2020, Elsevier.

Yu et al. reported the successful preparation of well‐crystalline graphite on a Ni cathode by a “dissolution‐precipitation” process (Figure 5A).^[^ ^26^ ^]^ During the electrolysis, the dissolved CO_3_^2−^ can be deposited as amorphous carbon at the electrode, which then starts to diffuse into the Ni cathode at the high working temperature (650°C). The continuous carbon diffusion finally leads to the oversaturation of the carbon atoms in Ni cathode, enabling the precipitation of graphene layers. Carbon materials with ultra‐high graphitization degree can be achieved by matching the applied current density with the diffusion of carbon atoms into Ni electrode. As shown in Figure 5B, the *I*_D_/*I*_G_ ratio is only 0.09 at 650°C and a current density of 5 mA/cm^2^. Note that *I*_D_ and *I*_G_ refer to the intensity of D and G band in the Raman spectrums, respectively. The D band at 1350 cm^−1^ is related to the edge‐induced disorder in graphite, while the G band at 1580 cm^−1^ is related to the sp^2^ vibrations from perfect graphitic structures. Smooth carbon layers after electrolysis can be observed at current densities of 5 and 10 mA/cm^2^ (Figure 5C,D). The graphitization degree of the carbon products can be further improved (*I*_D_/*I*_G_ ratio: 0.07) by increasing temperature to 750°C. Besides, the mixed nickel oxide/tricobalt tetroxide  cathode can serve as catalyst for promoting the graphitization.^[^ ^24^ ^]^ Under a cell voltage of 2.1 V, a graphite‐encapsulated nickel‐cobalt (NiCr) alloy nanocomposite can be obtained owing to the in situ catalytic graphitization of the reduced NiCo alloy.

Moreover, it has been reported that the addition of Li_2_SO_4_ in Li_2_CO_3_─Na_2_CO_3_─K_2_CO_3_ can strongly promote the graphitization of the carbon products.^[^ ^37^ ^]^ During electrolysis, the added SO_4_^2‐^ ions are reduced into sulfur (S) and sulphide ions (S^2−^ ), which will further react with imperfect carbon to form carbon disulfide (CS_2_) and sulfur dioxide (SO_2_) gases, respectively. A higher graphitization degree of carbon is achieved by the removal of imperfect carbon atoms. Additionally, the graphitization degree of carbon products can be enhanced by elevating the working temperature. The *I*_D_/*I*_G_ ratio decreases from 0.83 to 0.3 as the temperature increases from 475°C to 825°C, as shown in Figure 5E.

In general, the electroreduction of CO_2_ into carbon in Na_2_CO_3_─K_2_CO_3_ eutectic is thermodynamically unfavorable because of its high decomposition potential (Figure 1A). However, with the addition of sodium vanadate (NaVO_3_) into molten Na_2_CO_3_─K_2_CO_3_, large amounts of graphitic carbons are obtained.^[^ ^78^ ^]^ The graphitization degree of the obtained carbon materials increased with the increasing NaVO_3_ concentration, the applied cell voltage, and the electrolysis time (Figure 5G). The *I*_D_/*I*_G_ ratio of the carbon products is 0.489 after electrolysis at a cell voltage of 4.0 V for 10 h in the 15 wt% NaVO_3_ salt. The authors proposed an interesting “homogeneous catalytic” mechanism to explain this special phenomenon, as shown in Figure 5F.^[^ ^102^ ^]^ During the electrolysis, V_2_O_7_^4−^ ions are first electro‐reduced to vanadium trioxide (V_2_O_3_), followed by the co‐electroreduction of the V_2_O_3_ and CO_3_^2−^ ions to form graphitic carbon and regenerate V_2_O_7_^4−^ ions, resulting in the continuous conversion of CO_3_^2−^ ions into graphitic carbons.

### 2.3 Preparation of graphitic carbons in molten chlorides

The deposited carbon products in molten chlorides highly depend on the salt composition. In most cases, the carbon products are amorphous in LiCl‐based chlorides.^[^ ^52^, ^53^, ^56^, ^91^ ^]^ In contrast, various graphitic materials, such as graphite sheet, graphene, CNTs, and CNFs, can be achieved in CaCl_2_‐based molten chlorides, which might be attributed to the high working temperature.^[^ ^27^, ^28^, ^29^, ^30^, ^31^, ^32^, ^33^, ^34^, ^103^ ^]^

**FIGURE 6:**
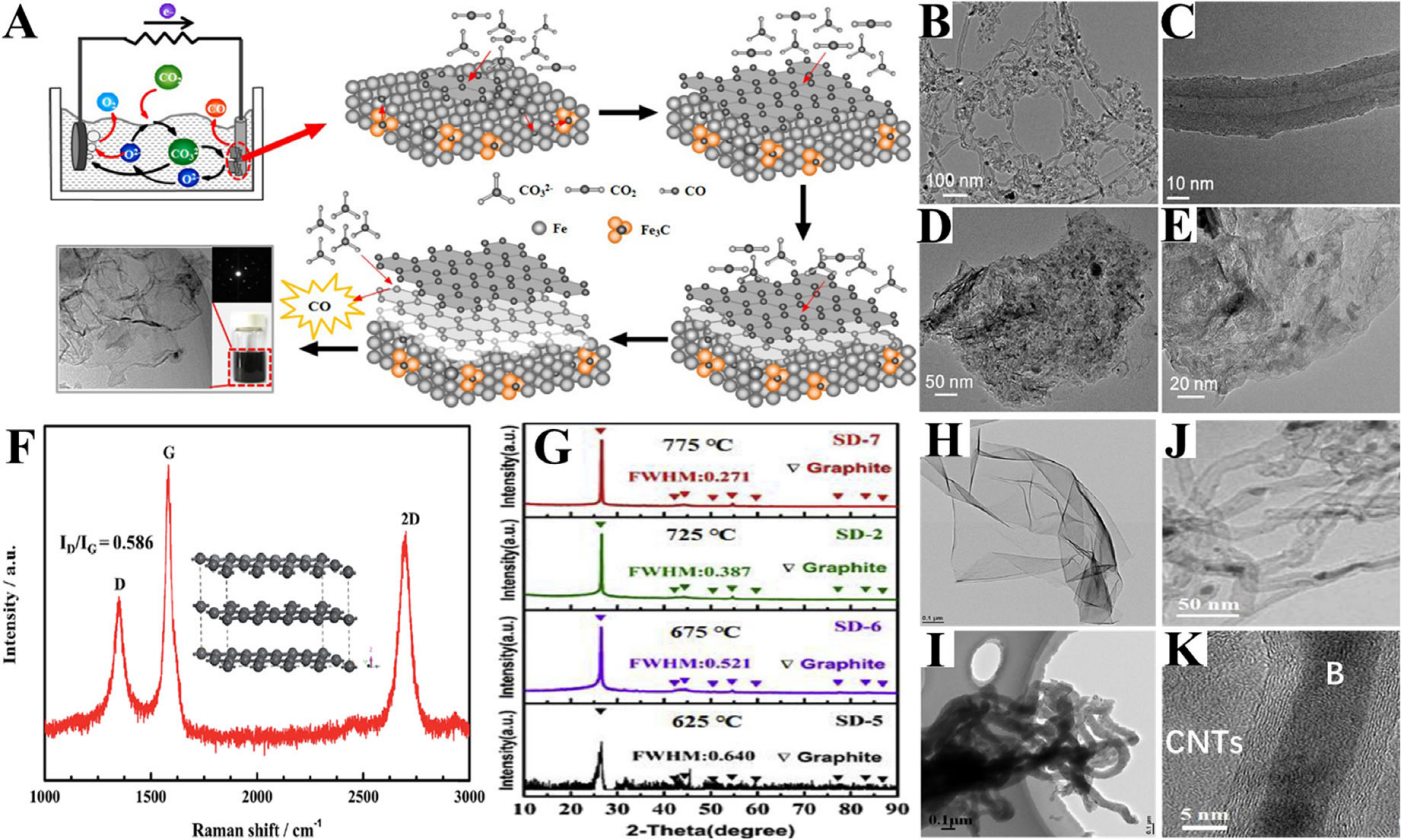
(A) Schematic illustration of electrochemical extraction of graphene on a stainless‐steel cathode in molten CaCl_2_─NaCl─CaO. Reproduced with permission.^[^ ^28^ ^]^ Copyright 2016, Wiley‐VCH. (B–E) The TEM images of obtained CNTs (at 750°C) and graphite sheets (at 850°C) on a glass carbon cathode in molten CaCl_2_─NaCl─CaO. Reproduced with permission.^[^ ^29^ ^]^ Copyright 2017, Royal Society of Chemistry. (F) Raman spectra of the graphite sheet obtained in CaCl_2_─CaO. Reproduced with permission.^[^ ^27^ ^]^ Copyright 2015, Royal Society of Chemistry. XRD patterns (G) and TEM images (H,I) of the obtained carbon products in CaCl_2_─NaCl─Li_2_SO_4_─CaO. Reproduced with permission.^[^ ^30^ ^]^ Copyright 2019, Elsevier. (J,K) The TEM images of the CNTs filled with amorphous boron in molten CaCl_2_─CaCO_3_─B_2_O_3_. Reproduced with permission.^[^ ^32^ ^]^ Copyright 2018, The Electrochemical Society.

Ultra‐thin graphite sheets could be successfully synthesized in molten CaCl_2_─CaO at a temperature of 850°C using a stainless steel cathode, as verified by Raman, X‐ray diffraction (XRD), and transmission electron microscopy (TEM).^[^ ^27^ ^]^ However, the formation mechanism of these graphitic materials is not fully understood. The formation of graphitic carbons could be related to either the high working temperature or the catalytic effect from the cathode. Further studies demonstrated that the cathode could have a significant influence on the produced products. In molten CaCl_2_─NaCl─CaO, the application of a stainless‐steel cathode led to the production of graphene at 750°C.^[^ ^28^ ^]^ It was proposed that the deposited C would react with the stainless steel to form the Fe_3_C catalyst, leading to the formation of graphite layers and the subsequent exfoliation by CO gas to generate graphene (Figure 6A,F). With the use of a glass carbon cathode in molten CaCl_2_─NaCl─CaO, the carbon products are mainly comprised of CNTs at 750°C (Figure 6B,C), while graphite sheets are observed at 850°C (Figure 6D,E).^[^ ^29^ ^]^ However, carbon products consisting of amorphous spherical carbons are achieved with a graphite cathode in molten CaCl_2_─NaCl─CaO at 750°C. The reason for this difference has not been revealed yet.

Carbon material with high graphitization degree could be prepared by the direct addition of Li_2_SO_4_ into CaCl_2_‐based chlorides at a temperature of 675°C.^[^ ^30^, ^31^ ^]^ The introduction of sulfur into carbon matrix greatly increases the graphitization degree, especially at high working temperatures (Figure 6G). The carbon products are mainly comprised of graphite flakes and CNFs (Figure 6H,I). Moreover, by employing a pulse‐current technique, well‐crystallinity of graphite nanostructures can be obtained in CaCl_2_─NaCl─LiCl─CaO eutectic.^[^ ^103^ ^]^ Besides, the addition of boron oxide (B_2_O_3_) in CaCl_2_─CaCO_3_ results in Boron‐filled CNTs (Figure 6J,K).^[^ ^32^ ^]^

## 3 ELECTROCHEMICAL GRAPHITIZATION OF AMORPHOUS CARBONS

Small amounts of catalysts or additives are usually needed for the electrochemical conversion of CO_2_ into graphitic carbons or doped graphitic carbons in molten salts, which may contaminate the carbon products. Recently, a molten salt electrochemical graphitization strategy has been proposed for the transformation of amorphous carbons (e.g., carbon black and carbon fiber) into graphitic carbon materials without using any catalysts.^[^ ^38^, ^39^, ^104^ ^]^ This strategy can be further utilized for the graphitization of low‐value solid carbon wastes (inferior coal and biomass).^[^ ^105^, ^106^ ^]^

### 3.1 Basic principle of electrochemical graphitization

As is well known, the presence of oxygen in the amorphous carbons will hinder the graphitization process and only can be removed at temperatures >1300°C together with other volatile residues.^[^ ^1^ ^]^ Inherited the electro‐deoxidation of metal oxides from the FFC Cambridge Process, Peng et al. successfully accomplished the electrochemical graphitization of amorphous carbons in molten CaCl_2_ at a relatively low temperature of 850°C.^[^ ^37^, ^104^ ^]^

**FIGURE 7:**
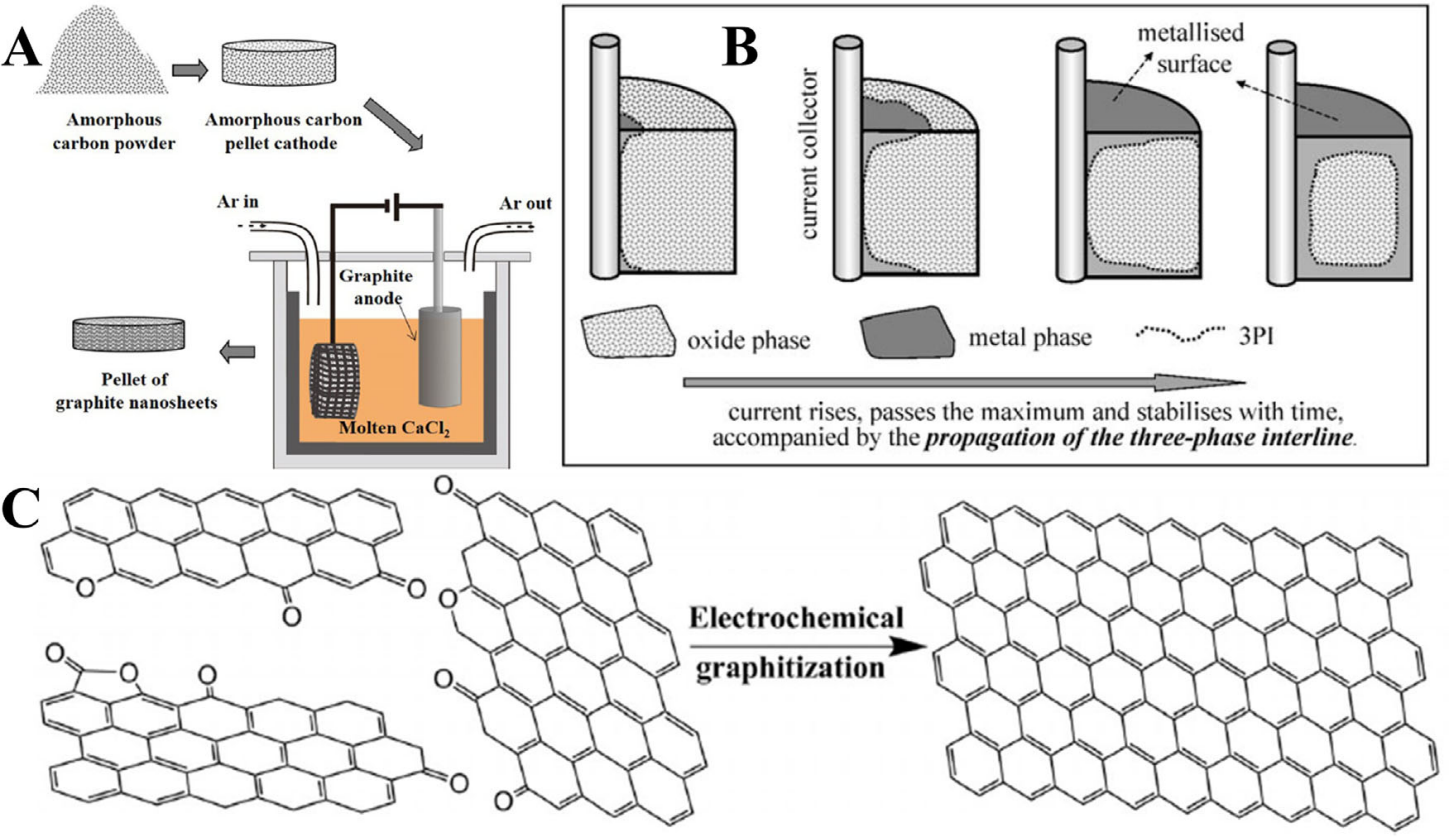
(A) Schematic illustration of the electrochemical graphitization of amorphous carbons in molten CaCl_2_. Reproduced with permission.^[^ ^38^ ^]^ Copyright 2017, Wiley‐VCH. (B) Schematic illustration of the 3PI propagation for the electrochemical reduction of an oxide pellet. Reproduced with permission.^[^ ^107^ ^]^ Copyright 2004, Springer Nature Switzerland AG, Part of Springer Nature. (C) Schematic illustration of the electrochemical graphitization of amorphous carbon into graphite. Reproduced with permission.^[^ ^38^ ^]^ Copyright 2017, Wiley‐VCH.

As shown in schematic illustration (Figure 7A), the electrolysis is conducted between the amorphous carbon cathode and a graphite anode.^[^ ^38^ ^]^ This electrochemical graphitization process involves two steps, the electrochemical removal of oxygen through cathodic polarization and the rearrangement of carbon atoms into a graphite crystal lattice. The electro‐deoxidation process of amorphous carbons basically follows a “three‐phase interline” (3PI) propagation mechanism (Figure 7B).^[^ 107, 108, 109 ^]^ The electro‐deoxidation step first proceeds at the current collector/amorphous carbon/electrolyte interline. At the initial stage, the surface amorphous carbon that is attached to the current collector will be electro‐reduced and subsequently converted into graphite, which has the same effect as increasing the 3PI interline. The 3PI propagates along the surface of the pellet with an appreciable speed due to the rapid dissolution of the surface oxygen, while the 3PI propagation speed of the 3PI into pellet becomes slower with a longer diffusion distance for oxygen to migrate. After the removal of oxygen in amorphous carbon, the disordered carbon atoms spontaneously rearrange into highly graphitized carbon materials (Figure 7C).^[^ ^38^ ^]^

### 3.2 Amorphous carbon feedstocks

**FIGURE 8:**
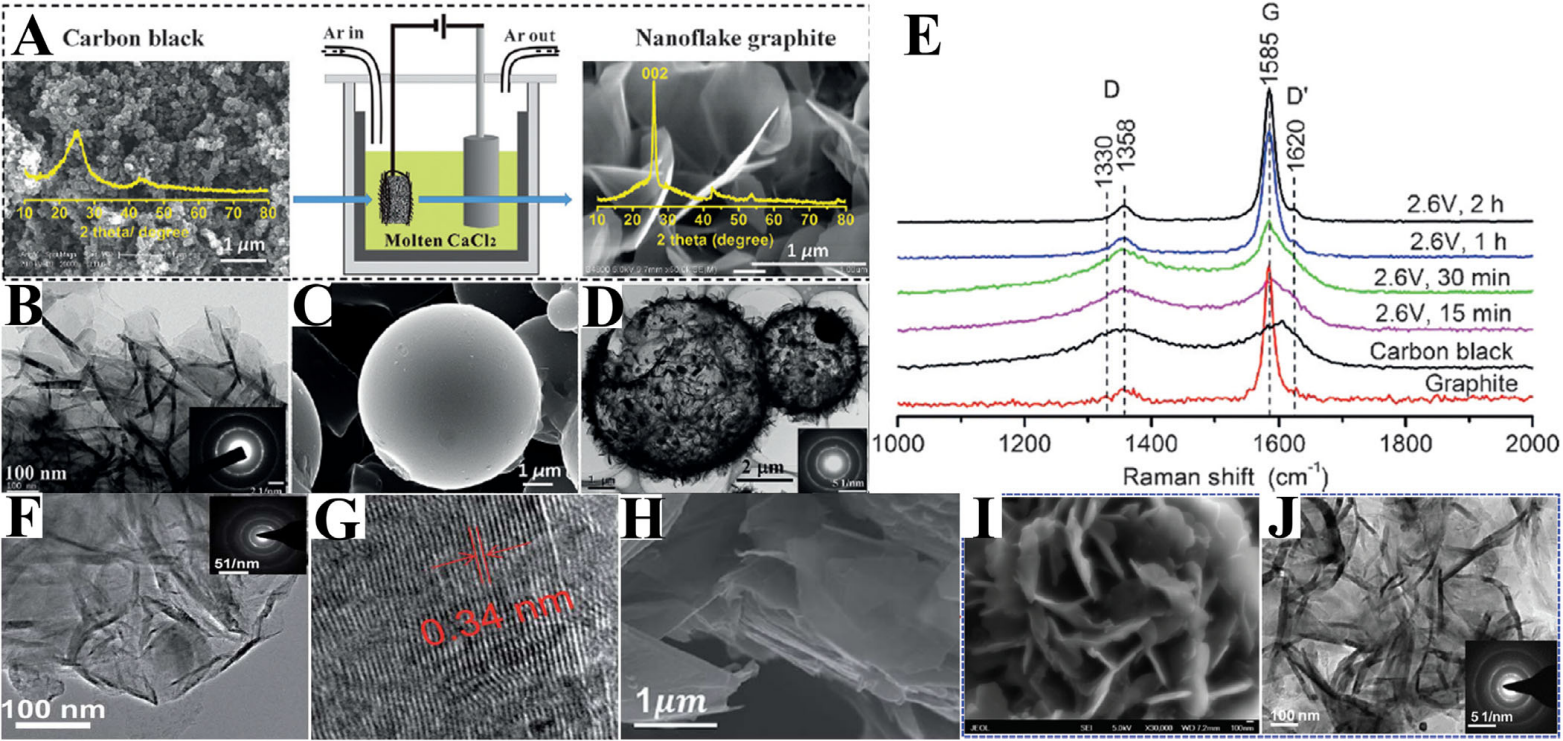
(A) Schematic illustration of the electrochemical graphitization process in molten CaCl_2_; (B) TEM image of the reduced carbon black; SEM images of the carbon microsphere before (C)and after graphitization (D); Raman spectrums (E) of carbon black under various electrolytic conditions. Reproduced with permission.^[^ ^37^ ^]^ Copyright 2017, Wiley‐VCH. (F,G) TEM images of the cellhouse‐derived flake graphite. Reproduced with permission.^[^ ^105^ ^]^ Copyright 2019, Royal Society of Chemistry. (H) SEM image of interior coal‐derived flake graphite. Reproduced with permission.^[^ ^106^ ^]^ Copyright 2019, Royal Society of Chemistry. (I,J) SEM and TEM images of the amorphous carbon‐derived “flower‐like” graphite. Reproduced with permission.^[^ ^104^ ^]^ Copyright 2019, Royal Society of Chemistry.

The electrochemical graphitization process has been demonstrated to be applicable to various amorphous carbon feedstocks, from “soft carbons” to “hard carbons.” The typical “hard carbon,” carbon black, which are non‐graphitizable by the conventional high‐temperature treatment, were successfully converted into nanoflake graphite by cathodic polarization under a cell voltage of 2.6 V for 2 h (Figure 8A,B).^[^ ^37^ ^]^ The *I*_D_/*I*_G_ ratio of the carbon products is calculated to be 0.11, close to that of commercial graphite. The Raman results show the gradual graphitization of carbon black during the electrolysis (Figure 8E). The dense carbon micro‐spheres could also be converted into a porous shell structure (Figure 8C,D). Moreover, the solid carbon wastes with high carbon content, such as biomass, have been demonstrated to be transformed into highly graphitized carbon materials using this strategy (Figure 8F,G).^[^ ^105^ ^]^ Zhu et al. reported the electrochemical graphitization of inferior coals into graphite flakes in molten CaCl2 at 2.6 V and 950°C (Figure 8H).^[^ ^106^ ^]^ Tu et al. demonstrated the electrochemical conversion of amorphous carbon into graphite in CaCl_2_─LiCl salt (Figure 8I,J).^[^ ^104^ ^]^

In this electrochemical graphitization process, the applied cell voltage can have a huge influence on the graphitization process. Generally, a high cell voltage facilitates the graphitization process. However, it should be limited to prevent the formation of CaC_2_. It has been reported that in CaCl_2_ with a graphite anode, the electrolysis at 2.6 V for 2 h leads to a graphite recovery of 80%. When the cell voltage is increased to more than 2.7 V, the carbon recovery will decrease to 50.9%. Further increase in cell voltage causes severe mass loss of carbon (as low as 5.2%).^[^ ^37^ ^]^ In addition, the value of cell voltage also depends on the anode material, inert anode usually requires a higher cell voltage than that of graphite due to its relatively low conductivity. A cell voltage of 3.0 V for SnO_2_ anode is tested to be effective for electrochemical graphitization.^[^ ^104^ ^]^

The electrolysis time for completing the graphitization process strongly depends on the raw carbon precursors. The carbon feedstocks with high‐content impurities (e.g., biomass and inferior coals) require a much longer electrolysis time than that of carbon black or carbon fibers. An electrolysis time of 2 h is enough for the graphitization of carbon black, while the time for biomass could be 8 h. It should be noted that, the obtained graphitic carbons almost possess the same sizes as their precursors, suggesting that the electrochemical graphitization begins at the solid carbon/electrolyte interface, as shown in Figure 8C,D. The electrochemical graphitization of carbon fibers leads to the formation of graphite pipes.^[^ ^37^ ^]^ And the addition of Ni or Co catalyst results in the formation CNTs.^[^ ^105^ ^]^

In summary, a simple and facile electrochemical strategy for conversion of amorphous carbon into graphite is demonstrated at low temperatures without using any catalysts. This process is preliminary studied, and more work is needed to reveal the graphitization mechanism. Moreover, the present studies focus on the graphitization of high carbon content materials (>90%), more attention should be paid to the graphitization of carbon feedstocks containing higher impurities (including but not limited to oxygen (O), phosphorus (P), sulfur (S), nitrogen (N), and hydrogen (H)). The detailed investigations on these carbon precursors as well as the corresponding graphitization mechanisms could lead to new discoveries in this field. Furthermore, the purity and graphitization degree of the obtained carbon materials should be improved for practical applications.

## 4 ENERGY STORAGE APPLICATIONS OF GRAPHITIC CARBONS

Carbon materials in modern society have been widely applied for energy storage and conversion due to its conductivity, chemical stability, availability, and variety.^[^ ^5^, ^6^, ^9^ ^]^ In particular, the graphitic carbon materials prepared in molten salts exhibit excellent electrochemical performance when tested as electrode material for energy storage (e.g., batteries and supercapacitors.^[^ ^31^, ^37^, ^39^, ^63^, ^104^, ^105^, ^106^ ^]^

**FIGURE 9:**
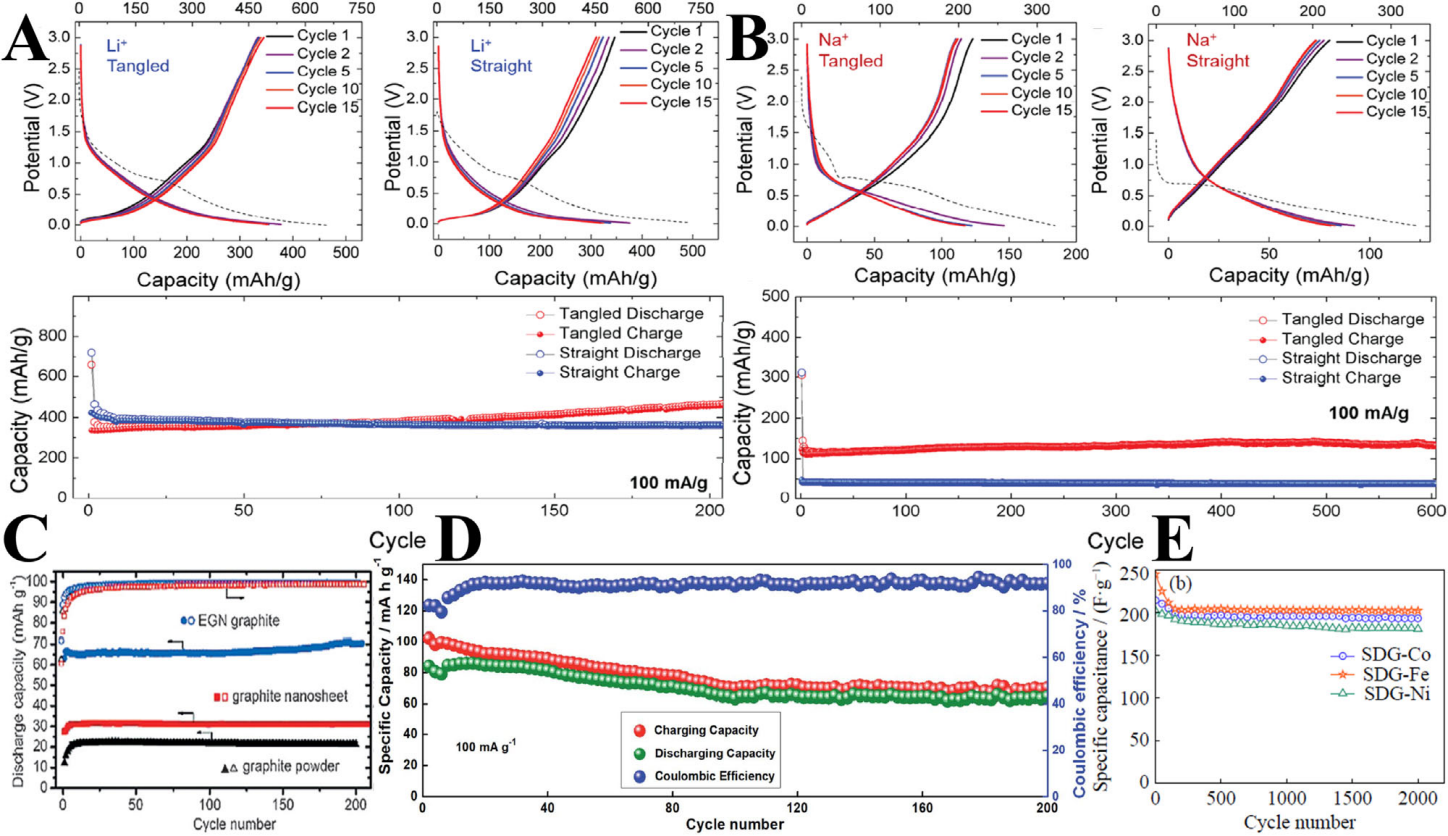
(A,B) The CO_2_‐derived straight and tangled CNTs as anode materials for Li‐ion and Na‐ion batteries. Reproduced under the terms of the ACS AuthorChoice License.^[^ ^63^ ^]^ Copyright 2016, American Chemical Society. (C) The capacity of electrochemically generated nano‐graphite through cathodic polarization for Li‐ion batteries. Reproduced with permission.^[^ ^37^ ^]^ Copyright 2017, Wiley‐VCH. (D) The capacity of cellhouse‐derived flake graphite for Al‐ion batteries. Reproduced with permission.^[^ ^105^ ^]^ Copyright 2019, Royal Society of Chemistry. (E) The specific capacitance of S‐doped CNFs for supercapacitors at 1.25 A/g. Reproduced with permission.^[^ ^31^ ^]^ Copyright 2020, University of Science and Technology Beijing and Springer‐Verlag GmbH Germany, Part of Springer Nature.

Licht et al. have tested the storage capability of both straight and tangled CNTs (derived from the electroreduction of CO_2_ in molten Li_2_CO_3_) as anode materials in lithium‐ion (Li‐ion) batteries and sodium‐ion (Na‐ion) batteries (Figure 9A,B).^[^ ^63^ ^]^ For the Li‐ion battery, the reversible capacity of the straight and tangled CNTs stabilizes at ∼350 mAh/g after 15 cycles with a high coulombic efficiency near 100%. In the long‐term cycling tests, while the straight CNTs (less disordered) remain at a stable capacity, the capacity of the tangled CNTs (more defects) gradually increases to 460 mAh/g after 200 cycles at 100 mA/g. In the case of Na‐ion battery, the tangled CNTs exhibit reversible capacities ∼135 mAh/g, two times that of the straight CNTs. The results show the defects in a CNT material are of great significance to activate the mechanism for Li^+^ and Na^+^ storage.

The graphitized carbons after electrochemical graphitization of amorphous carbon black in CaCl_2_ are also tested as anode material for Li‐ion batteries.^[^ ^37^ ^]^ This electrochemically generated graphite material shows a capacity of 65 mAh/g at 1800 mA/g over 200 cycles (Figure 9C), while the capacity of commercial graphite nanosheet is 31 mAh/g. Zhu et al. tested the interior coal‐derived flake graphite for Li‐ion battery, and a stable capacity of 340 mAh/g at 2C after 1000 cycles is achieved, superior to that of commercial graphite.^[^ ^106^ ^]^ The cellulose‐derived graphitic carbons are tested for Al‐ion batteries (Figure 9D), which deliver a capacity of 60 mAh/g at 100 mA/g after 200 cycles.^[^ ^105^ ^]^ Besides, S‐doped CNFs obtained by electrochemical reduction of CO_2_ in molten CaCl_2_─NaCl─Na_2_SO_4_ are measured as electrode material for supercapacitors.^[^ ^31^ ^]^ The specific capacitances (Figure 9E) of the CNFs calculated at 0.5, 1.25, 2.5, 5, and 10 A/g were 348.5, 244.25, 214.5, 159, and 128 F/g, respectively, showing excellent electrochemical performance. These studies demonstrate this electrochemical graphitization route can be an effective approach to prepare graphitic carbons for high‐performance batteries and supercapacitors.

## 5 ENERGY CONSUMPTION AND COST ESTIMATION

The scale‐up of molten electrochemical approach for producing graphitic carbons lays on the energy consumption of the process and the value of the carbon products. Therefore, it is necessary to evaluate the energy consumption and costs of all the electrochemical synthesis routes for further large‐scale production. For the electrochemical transformation of CO_2_ into graphitic carbons, the energy consumption of the electrolysis for carbon deposition from CO_2_ could be calculated by the following equation:

(14) $$P=\frac{8.933E_{\mathrm{cell}}}{\eta }$$

where *P* is the energy consumption for depositing 1 kg carbon, kWh/ kg C; *E*_cell_ is the cell voltage applied during the electrolysis; *η* is current efficiency. The energy consumption can vary significantly, depending on the salt system, as summarized in Tables 1 and 2.

In molten Li_2_CO_3_, the cell voltage ranging from 0.9 to 1.4 V at 770°C is needed to drive the electrolysis, and CNFs (or CNTs) are produced with a current efficiency of 80%–100%, resulting in an energy consumption ranging from 8.04 to 15.63 kWh/kg C. The energy cost at $0.1 per kWh is equivalent to $800 to $1560 for producing one ton of CNFs (CNTs). Considering the additional costs of the electrodes, Li_2_CO_3_ salt, and ancillary equipment, a combined additional cost is ∼$2140 per ton CNTs. These costs are much lower than the costs of carbon products produced by traditional CVD process ($25,000 per ton CNFs or $200,000–$400,000 per ton CNTs).^[^ ^62^, ^63^, ^102^ ^]^ Note that the cost of molten salt could be further reduced by the introduction of inexpensive Na_2_CO_3_.

The minimum energy consumption for carbon deposition in molten Li_2_CO_3_─Na_2_CO_3_─K_2_CO_3_ and CaCl_2_─NaCl─CaO is reported to be 35.59 kWh/kg C and 44.06 kWh/kg C, respectively.^[^ ^53^, ^54^ ^]^ The corresponding energy cost in the systems, taking molten Li_2_CO_3_─Na_2_CO_3_─K_2_CO_3_ as an example, is $3560 per ton CNTs (at $0.1 per kWh), which causes a total cost of $5937 per ton CNTs according to the calculation from ref. [21]. In addition, it has been reported that the energy consumption of the electrochemical graphitization of amorphous carbon in molten CaCl_2_ is in the range of 4.5 to 5.5 kWh/kg C, which is much lower than that from traditional high‐temperature process (32.1 kWh/kg C).^[^ ^37^, ^104^ ^]^

The low cost of this molten salt electrochemical strategy for CNT or CNF production implies a significant economic incentive. However, the energy consumption for sustaining the high working temperature is not considered in the calculations, which may underestimate the overall costs. Moreover, the yield and purity of the deposited CNT (or CNF) as well as the precise control of its growth needed to be further improved to meet the industry standard. Also, from the view of mitigating global warming, the electrochemical conversion of CO_2_ into carbon material should be powered by renewable energies such as solar and wind, complicating the design of cell system.^[^ ^44^, ^102^ ^]^

## 6 SUMMARY AND PERSPECTIVE

The traditional process for synthetic graphite usually requires a temperature close to 3000°C, thus a technical methodology that can effectively produce graphite at low temperature is of great importance. Molten salt electro‐preparation of graphitic carbons has been demonstrated to be a promising method due to its simplicity, high‐efficiency, tunability, and compatibility. Typically, the carbon precursors, such as CO_2_ and amorphous carbons, can be converted into graphitic materials in molten salts. In general, CO_2_ gas can be directly or indirectly (reduction of CO_3_^2−^ ions) reduced into carbon products. To inhibit the competing CO evolution reaction, a low temperature is suggested for carbon deposition. However, the carbon products are mainly comprised of amorphous carbon in molten carbonates or chlorides.

With the addition of catalysts or other additives, high‐quality graphitic carbons can be achieved by controlling the electrolytic condition, such as the concentration of catalyst, the treatment of electrode, and the control of current density. High quality CNT (or CNF) products with controllable properties can be achieved under the catalytic effect of the deposited Ni (or Fe) metal. The CNT (or CNF) product can be also tuned by doping non‐metal elements (e.g., N, B, and P). Besides, carbon materials with high graphitization degree, such as graphite flake, can be achieved through direct cathodic reduction. Furthermore, these graphitic carbon materials present excellent performance when used as electrode materials for energy storage (e.g., batteries and supercapacitors). More importantly, the cost of carbon deposition using this approach is much lower than that of carbon produced from the traditional process, showing great potential for large‐scale graphite production.

While this technology shows great promise for commercialization, it is important to pay attention to some issues that have not been fully addressed. Understanding the mechanism of the reduction of CO_2_ in molten salt is a remaining challenge. Very limited information for CO_2_ reduction can be in situ obtained by the present electrochemical techniques (e.g., CV and SWV tests), and other ex situ techniques, such as SEM and XRD, are required for further revealing the electrochemical mechanism. However, it is of great importance to real‐time monitor the state change of the reactants to be studied under a current (or potential) excitation, which can provide valuable insights (e.g., the detection of intermediate CO_2_^2−^ ions). Thus, the development of in situ techniques designed for high temperature molten salts, such as in situ high temperature Raman and computed tomography are urgent for the molten salt electrochemistry.

The purity and size control as well as the crystallinity of the graphitic carbons still needs to be further improved. In the process of electro‐reduction of CO_2_, trace amounts of catalysts are needed for the formation of graphitic carbons, which may contaminate the final products. Also, the diameter of CNTs increases with the flowing charge. The uneven current distribution at the cathode can result in the uneven growth of CNT products. To date, the investigation on the optimization of electrode design has not been conducted yet. Besides, the inert anodes for molten salts were only demonstrated to stable in short‐term tests (several days). Long‐term stability of these inert anodes in molten salts should also be considered for practical applications. In addition to these current issues, more efforts should be put to explore the synthesis of new carbon nanomaterials and the defect engineering for carbon deposits as electroactive materials. Until now, no scale‐up experiments for either electro‐reduction of CO_2_ or the electrochemical graphitization process are reported. From the point of view of engineering, the technical analysis of the cell stability, product separation and collection, as well as mass and energy balance is still lacking. But with the application of carbon tax and the fast development of clean energy, the large‐scale tests might be accomplished in near future.

## CONFLICT OF INTEREST

The authors declare no conflict of interest.
